# Patient- and cycle-specific factors affecting the outcome of frozen–thawed embryo transfers

**DOI:** 10.1007/s00404-023-07019-3

**Published:** 2023-04-16

**Authors:** Verena Holschbach, Hannah Kordes, Jens Erik Dietrich, Thomas Bruckner, Thomas Strowitzki, Ariane Germeyer

**Affiliations:** 1 Department of Gynecologic Endocrinology and Fertility Disorders, Heidelberg University Hospital, INF440, 69120 Heidelberg, Germany; 2 Institute of Medical Biometry and Informatics, University of Heidelberg, INF 130, 69120 Heidelberg, Germany

**Keywords:** FET, Cryopreservation, IVF/ICSI, Prognostic factor, Endometrial preparation

## Abstract

**Purpose:**

This study attempted at identifying the main parameters influencing the outcome of frozen embryo transfers.

**Methods:**

This is a single-center retrospective cohort study of 830 frozen-embryo-transfer cycles performed at a German university hospital from January 2012 to December 2016. Main outcome parameters were the clinical pregnancy and live birth rate. Twelve patient- and cycle-dependent factors were analyzed in terms of their influence on the outcome of frozen embryo transfers. Multivariate logistic regression analysis was used for the modelling of the dependency of the different parameters on outcomes.

**Results:**

The clinical pregnancy rate in our study was 25.5%, the live birth rate was 16.1% with an average maternal age of 34.2 years at the time of the oocyte retrieval. In the univariate analysis age, number of transferred embryos, blastocyst versus cleavage stage transfer, embryo quality and mode of endometrial preparation affected the birth rate significantly. The birth rate after artificial endometrial preparation was significantly lower than the birth rate after transfers in modified natural cycles (12.8 versus 20.6% with *p* = 0.031). The multivariate logistic regression analysis showed a significant independent influence of age, number of transferred embryos, culture duration and mode of endometrial preparation on the frozen embryo transfer success rates. Body mass index, nicotine abuse, a history of PCO syndrome or endometriosis and the co-transfer of a second poor-quality embryo to a good-quality embryo appeared to be irrelevant for the outcome in our collective.

**Conclusion:**

Age, number of transferred embryos, embryo culture duration and the mode of endometrial preparation are independent predictive factors of frozen embryo transfer outcomes.

## What does this study add to the clinical work

**Table:** 

Age, number of transferred embryos, embryo culture duration and the mode of endometrial preparation are independent predictors of the outcome of frozen embryo transfer cycles.	

## Introduction

In Europe, every year approximately 2–6% of all children born were conceived by artificial reproductive techniques [1]. Since the report of the first pregnancy achieved by transfer of a cryopreserved embryo in 1983 [2] frozen–thawed embryo transfer (FET) has become one of the most important techniques of medically assisted reproduction (MAR) [3, 4]. The influencing factors of IVF and ICSI have been studied extensively, but less data are available about key factors for FET. Although there have been investigations of FET in America [5], the Netherlands [6], Australia [7], Finland [8] and China [9], these results cannot be simply transferred to MAR units in Germany where legal restrictions regulate the use of assisted reproductive technologies [10]. Several previous studies have analyzed the effect of single parameters such as age, embryo quality, blastocyst culture, endometrial preparation, or endometrial thickness [11–16]. Only few studies examined the interrelation of these numerous influencing factors by multivariate logistic regression analysis [6, 8, 17]. These studies had controversial results.

Using multivariate logistic regression analysis, this study tries to identify the main parameters influencing the outcome of frozen embryo transfers to optimize reproductive treatment in German fertility clinics.

## Material and methods

### Study design

This is a unicentric retrospective cohort study of all FET cycles performed at the Heidelberg university hospital from January 1, 2012 to December 31, 2016. In this period 830 FET cycles were initiated. Only the first FET cycle of a couple was considered for analysis. Cycles with maternal age older than 42 years, triple embryo transfers and missing pregnancy test results were excluded. Of these FET cycles 122 cycles had to be cancelled before the transfer (due to degeneration of PN cells/embryos or abnormal preimplantation genetic testing findings). Finally, 431 cycles remained for analysis.

The following cycle data were documented: Age (years at the time of the oocyte retrieval and years at the time of the transfer), BMI (kg/m^2^), nicotine abuse at the time of the oocyte retrieval, previous history of endometriosis and PCO syndrome, number of previous IVF treatments, protocol for endometrial preparation, endometrial thickness (maximum diameter in mm), number of transferred embryos, quality of transferred embryos and embryo culture duration (days). Cycles after January 2017 were not included into the study collective as the embryo culture media was changed at this time.

### IVF treatment and cryopreservation protocols

Controlled ovarian stimulation and IVF/ICSI protocols haven been described previously [18]. According to the German Embryo Protection Act a limited number of PN oocytes is allowed to be cultured up to the blastocyst stage. Supernumerary fertilized PN stage oocytes or unintentionally developed blastocysts can be frozen. If more oocytes were fertilized than needed for transfer on day 2 or 3 or for a blastocyst culture, supernumerary PN cells were frozen according to the slow freezing protocol with the K-SICS-5000 Sydney IVF Cryopreservation Kit (Cook Medical, Bloomington, USA) or -from Oct 19 2016 on- with Freeze Kit Cleave (10166, Vitrolife, Sweden) in cryo tubes using a Biofreeze BV-65 (Consarctic, Westerngrund, Germany) and thawed with the K-SITS-5000 Sydney IVF Thawing Kit (Cook Medical, Bloomington, USA) or -from Oct 19 2016 on- Thaw Kit Cleave (10167, Vitrolife, Sweden).

MII oocytes, cleavage stage embryos and blastocysts were cryopreserved by vitrification using Kitazato Vitrification Media (91101 or 91171, Kitazato BioPharma Co, Tokyo, Japan) and the Kitazato Cryotop (open system). Warming was performed using Kitazato Thawing Media (91121 or 91182, Kitazato BioPharma Co, Tokyo, Japan).

The endometrium was prepared either by hormonal substitution (HRT-FET) or in a modified natural cycle (mNC-FET) with spontaneous follicle maturation followed by spontaneous or LH triggered ovulation and low dose luteal support with 200 mg progesterone. Indications for HRT cycles were anovulatory cycles, oligomenorrhea and amenorrhea.

For an intended blastocyst transfer up to 9 PN stage oocytes were thawed in order to culture up to 5 cells (in accordance with the German embryo protection act) or cryopreserved day 4 or 5 embryos were warmed and transferred the same day. In case of cryopreserved day 2 and 3 embryos, these were warmed and transferred on the same day without further culture.

### Outcome measures

The primary outcomes were clinical pregnancy and live birth rate. Definition of outcome measures:

Biochemical pregnancy: A serum HCG level of at least 10 IU/l 14 days after ovulation/13 days after start of progesterone in HRT cycles.

Clinical pregnancy: The presence of an intrauterine gestational sac at 6 weeks of gestation in the transvaginal ultrasound.

Live birth: Birth of at least one child after 24 + 0 weeks or with a birth weight of at least 500 g.

Miscarriage: Clinical pregnancies which did not continue to ongoing pregnancies. Twin pregnancies with vanishing of one twin were classified as ongoing pregnancies.

Implantation rate: Number of gestational sacs observed divided by the number of embryos transferred.

### Assessment of embryo quality

Embryo quality was determined daily according to the ESHRE Istanbul consensus [19]. For our study embryo quality was assessed at the day of transfer. It was retrospectively classified into 2 groups (1 = good-quality embryos = GQE), 2 = poor-quality embryo = PQE). Good-quality embryos were defined as 2–4-cell and grade A or B on day 2; 5–8-cell and grade A or B on day 3; 9–16-cell and grade A or B, compacting or fully compacted morula on day 4; blastocyst grade ≥ 3BB on day 5. Double embryo transfers were divided into three groups: (a) transfer of two good quality embryos, (b) transfer of a good- and poor-quality embryo and (c) transfer of two poor-quality embryos.

### Statistics

Statistical analysis was performed by SAS (SAS Institute, Cary, NC, USA) and SPSS (IBM SPSS Statistic, version 27.0, Armonk, NY, USA) in cooperation with the Institute of Medical Biometry and Informatics Heidelberg. Statistical significance level was set to *p* = 0.05. Confidence intervals were described as 95% intervals (95% CI). Significant differences between interval scaled parameters were calculated with *t* tests. Not normally distributed data were analyzed using Mann–Whitney-*U* test. Chi-Square tests were used for dichotomous traits. For the modelling of the dependency of the pregnancy outcome the multivariate logistic regression analysis was used.

## Results

Out of 431 FET 110 cycles led to a clinical pregnancy (clinical pregnancy rate = 25.5%, Table 1). Of these 110 clinical pregnancies 8 were lost to follow-up and 68 gave birth to a child (68/423; birth rate = 16.1%). 12 pregnancies were twin pregnancies (10.9% of the clinical pregnancies; 11 twin pregnancies after DET and 1 after SET), 7 patients gave birth to twins (10.3% of all births). The implantation rate per transferred embryo was 19.5% (Table 2).

**Table 1:** Outcome of 431 FET transfers (univariate analysis)

Factor	Total	Clinical pregnancy	No clinical pregnancy	*p * =	Total	Birth	No birth	*p * =
No of cycles	431	110 (25.5%)	321 (74.5%)	–	423	68 (16.1%)	355 (83.9%)	–
Mean age at oocyte collection (years) ± sdv	431	32.9 ± 3.9	34.7 ± 4.2	*p* < 0.0001	423	32.4 ± 3.8	34.6 ± 4.2	*p* < 0.0001
Mean age at transfer (years) ± sdv	431	33.9 ± 3.9	35.7 ± 4.2	*p* < 0.0001	423	33.5 ± 3.9	35.6 ± 4.2	*p* < 0.0001
Mean BMI ± sdv	431	23.6 ± 4.3	23.7 ± 4.2	*p* = 0.828	418	23.3 ± 4.2	23.7 ± 4.1	*p* = 0.498
Mean No of previous transfers ± sdv	431	2.2 ± 2.6	2.7 ± 2.5	*p* = 0.100	421	2.2 ± 2.8	2.6 ± 2.5	*p* = 0.236
Age < 35 years at oocyte collection	177	58 (32.8%)	119 (67.2%)	*p* = 0.004	207	43 (20.8%)	164 (79.2%)	*p* = 0.010
Age ≥ 35 years at oocyte collection	254	52 (20.5%)	202 (79.5%)		216	25 (11.6%)	191 (88.4%)	
Endometrial preparation: mNC	183	54 (29.5%)	129 (70.5%)	*p* = 0.103	180	37 (20.6%)	143 (79.4%)	*p* = 0.031
Endometrial preparation: HRT	248	56 (22.6%)	192 (77.4%)		243	31 (12.8%)	212 (87.2%)	
Age < 35 years at oocyte collection + mNC	113	33 (29.2%)	80 (70.8%)	*p* = 0.339	110	25 (22.7%)	85 (77.3%)	*p* = 0.460
Age < 35 years at oocyte collection + HRT	102	36 (35.3%	66 (64.7%)		97	18 (18.6%)	79 (81.4%)	
Age ≥ 35 years at oocyte collection + mNC	70	21 (30.0%)	49 (70.0%)	*p* = 0.004	70	12 (17.1%)	58 (82.9%)	*p* = 0.076
Age ≥ 35 years at oocyte collection + HRT	146	20 (13.7%)	126 (86.3%)		146	13 (8.9%)	133(91.1%)	
Mean EMR ± sdv	396	9.4 ± 1.7	9.4 ± 1.9	*p* = 0.938	388	9.4 ± 1.7	9.4 ± 1.9	*p* = 0.864
EMR < 8 mm	66	12 (18.2%)	54 (81.8%)	*p* = 0.123	66	7 (10.6%)	59 (89.4%)	*p* = 0.210
EMR ≥ 8 mm	330	90 (27.3%)	240 (72.7%)		322	54 (16.8%)	268 (83.2%)	
Age < 35 years at oocyte collection + EMR < 8 mm	36	6 (16.7%)	30 (83.3%)	*p* = 0.031	36	3 (8.3%)	33 (91.7%)	*p* = 0.054
Age < 35 years at oocyte collection + EMR ≥ 8 mm	168	59 (35.1%)	109 (64.9%)		160	36 (22.5%)	124 (77.5%)	
Age ≥ 35 years at oocyte collection + EMR < 8 mm	30	6 (20.0%)	24 (80.0%)	*p* = 0.912	30	4 (13.3%)	26 (86.7%)	*p* = 0.726
Age ≥ 35 years at oocyte collection + EMR ≥ 8 mm	162	31 (19.1%)	131 (80.1%)		162	18 (11.1%)	144(88.9%)	
SET	212	44 (20.8%)	168 (79.2%)	*p* = 0.026	209	24 (11.5%)	185 (88.5%)	*p* = 0.011
DET	219	66 (30.1%)	153 (69.9%)		214	44 (20.6%)	170 (79.4%)	
Age < 35 years at oocyte collection + SET	114	33 (28.9%)	81 (71.1%)	*p* = 0.294	111	19 (17.1%)	92 (82.9%)	*p* = 0.163
Age < 35 years at oocyte collection + DET	101	36 (35.6%)	65 (64.4%)		96	24 (25.0%)	72 (75.0%)	
Age ≥ 35 years at oocyte collection + SET	98	11 (11.2%)	87 (88.8%)	*p* = 0.008	98	5 (5.1%)	93 (94.9%)	*p* = 0.007
Age ≥ 35 years at oocyte collection + DET	118	30 (25.4%)	88 (74.6%)		118	20 (16.9%)	98 (83.1%)	
Transfer day 2–3	223	35 (15.7%)	188 (84.3%)	*p* < 0.0001	219	21 (9.6%)	198 (90.4%)	*p* = 0.0002
Transfer day 4–5	208	75 (36.1%)	133 (63.9%)		204	47 (23.0%)	157 (77.0%)	
DET: embryo quality 2 × good	129	47 (36.4%)	82 (63.6%)	*p* = 0.013	126	31 (24.6%)	95 (75.4%)	*p* = 0.030
DET: embryo quality 2 × poor	39	6 (15.4%)	33 (84.6%)		37	3 (8.1%)	34 (91.9%)	
Day 4–5 + SET: embryo quality 1 × good	84	27 (32.1%)	57 (67.9%)	*p* = 0.944	83	15 (18.1%)	68 (81.9%)	*p* = 0.949
Day 4–5 + DET embryo quality 1 × good + 1 × poor	16	5 (31.0%)	11 (69%)		16	3 (18.9%)	13 (81.3%)	
Smoker	60	18 (30.0%)	42 (70.0%)	*p* = 0.404	59	12 (20.3%)	47 (79.7%)	*p* = 0.3460
Non-smoker	369	92 (24.9%)	277 (75.1%)		362	56 (15.5%)	306 (84.5%)	
History of endometriosis	106	28 (26.4%)	78 (73.6%)	*p* = 0.833	104	20 (19.2%)	84 (80.8%)	*p* = 0.3255
No endometriosis known	323	82 (25.4%)	241 (74.6%)		317	48 (15.1%)	269 (84.9%)	
PCOS	55	16 (29.1%)	39 (70.9%)	*p* = 0.530	54	10 (18.5%)	44 (81.5%)	*p* = 0.6128
No PCOS	374	94 (25.1%)	280 (74.9%)		367	58 (15.8%)	309 (84.2%)

**Table 2:** Patient and cycle factors affecting the implantation rate (univariate analysis)

	*N* =	Implantation rate (%)	*p* value (CI)	< 35 years *N* =	Implantation rate (%) < 35 years	*p* value (CI)	≥ 35 years *N* =	Implantation rate (%) ≥ 35 years	*p* value (CI)
Total	431	19.5	(CI 16.1; 22.9)	215	25.8	(20.3; 31.3)	216	13.2	(9.3;17.1)
Endometrial preparation: mNC	183	23.5	*p* = 0.055 (− 0.1; 14.1)	113	25.2	*p* = 0.824 (− 1.3; 5.6)	70	20.7	*p* = 0.019 (1.9; 20.4)
Endometrial preparation: HRT	248	16.5		102	26.5		146	9.6	
Age at oocyte collection < 35 years	177	25.8	*p* < 0.001 (5.9; 19.4)	–	–	–	–	–	–
Age at oocyte collection ≥ 35 years	254	13.2		–	–	–	–	–	–
SET	212	21.2	*p* = 0.330 (− 3.5; 10.3)	114	29.8	*p* = 0.119 (− 2.2; 19.3)	98	11.2	*p* = 0.369 (− 11.5; 4.3)
DET	219	17.8		101	21.3		118	14.8	
Transfer day 2–3	223	10.1	*p* < 0.001 (− 26.2; − 12.8)	108	13.0	*p* < 0.001 (− 36.3; − 15;3)	115	7.4	*p* = 0.003 (− 20.4; − 4.4)
Transfer day 4–5	208	29.6		107	38.8		101	19.8	
DET: embryo quality 2 × good	129	20.9	*p* = 0.084 (− 1.3; 20.1)	67	24.6	*p* = 0.293 (− 9.7; 31.7)	62	16.9	*p* = 0.323 (− 6.2; 18.7)
DET: embryo quality 2 × poor	39	11.5		11	13.6		28	10.7	
EMR < 8 mm	66	13.6	*p* = 0.090 (− 16.0; 1.2)	36	13.9	*p* = 0.031 (− 26.8; − 1.3)	30	13.3	*p* = 0.927 (− 12.5; 11.4)
EMR ≥ 8 mm	330	21.1		168	28.0		162	13.9	
Smoker	60	21.7	*p* = 0.631 (− 12.3; 7.5)	37	20.3	*p* = 0.357 (− 7.8; 21.5)	23	23.9	*p* = 0.143 (− 28.2; 4.3)
Non-smoker	369	19.2		177	27.1		192	12.0	
History of endometriosis	106	20.8	*p* = 0.701 (− 1.6; 4.1)	61	25.4	*p* = 0.928 (− 11.6; 12.8)	45	14.4	*p* = 0.773 (− 11.2; 8.3)
No history of endometriosis	323	19.2		154	26.0		169	13.0	
PCOS	55	21.8	*p* = 0.624 (− 2.6; 5.2)	37	25.7	*p* = 0.982 (− 14.5; 14.8)	18	13.9	*p* = 0.722 (− 15.0; 13.7)
No PCOS	374	19.3		178	25.8		196	13.3	

### Univariate analysis

**Fig. 1:**
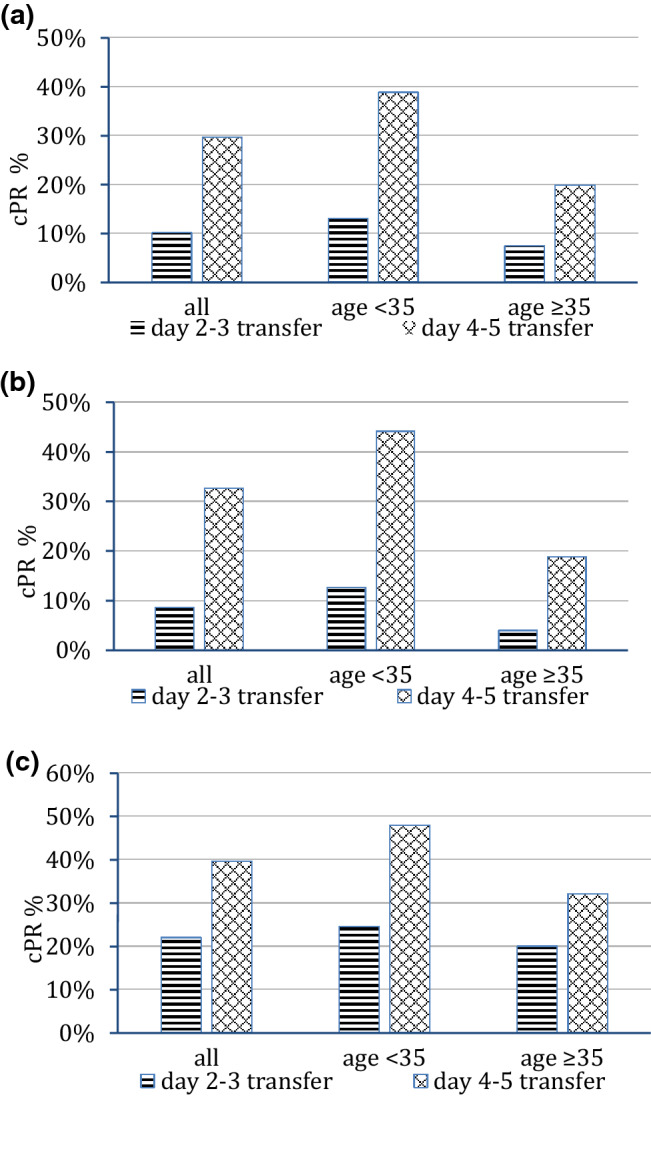
**a** Age dependent cPR after transfer on day 2–3 versus 4–5 (all). **b** Single embryo transfer only: age dependent cPR after transfer on day 2–3 versus 4–5. **c** Double embryo transfers only: age dependent cPR after transfer on day 2–3 versus 4–5

Patients with clinical pregnancies were significantly younger at the time of the oocyte collection than non-pregnant patients (Table 1, 32.9 vs 34.7 years, *p* < 0.0001). The clinical pregnancy rate was significantly higher after double embryo transfers compared to single embryo transfers (30.1% after DET vs 20.8% after SET) and after blastocyst culture with transfer on day 4–5 (36.1% after blastocyst culture vs 15.7% after day 2–3 transfer) (Table 1 and Fig. 1a–c).

**Fig. 2:**
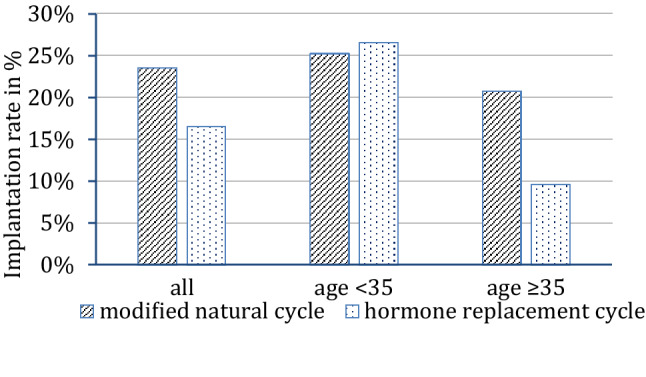
Age dependent implantation rates in mNC and HRT cycles

In cycles with DET the clinical pregnancy rate differed significantly as a function of the quality of the transferred embryos (36.4% after transfer of two good-quality embryos vs 15.4% after two poor-quality embryos). The age-stratified subgroup analysis comparing cycles with single and double embryo transfer showed that in patients younger than 35 years no significant difference of the outcome can be seen. A sonographic endometrial diameter of less than 8 mm led to a lower clinical pregnancy rate (27.3% with EMR of at least 8 mm vs 18.2% with EMR < 8 mm) but reached only in the subgroup of women younger than 35 years statistical significance. Here, an endometrial diameter of less than 8 mm correlated negatively with the clinical pregnancy rate (35.1% vs 16.7%, Table 1). In our collective, the lowest endometrial diameter with a live birth as outcome measured 6 mm. In the subgroup analysis of patients ≥ 35 years, FET in modified natural cycles led to significant higher clinical pregnancy rates in comparison to HRT cycles (30.0% versus 13.7%, Table 1, Fig. 2).

The effects of maternal age at the time of the oocyte retrieval, the number of transferred embryos, the mode of endometrial preparation and the culture duration were also reflected in the birth rates after FET. In cycles with blastocyst culture the co-transfer of a second embryo of poor quality to one good quality embryo did not improve the pregnancy or birth rate compared to a SET with a good quality embryo only (Table 1).

No significant difference for the BMI, nicotine abuse, a former diagnosis of endometriosis or PCO and the number of prior embryo transfers (fresh or frozen) was detected between successful FETs and FETs without clinical pregnancy or birth.

### Multivariate regression analysis

**Fig. 3:**
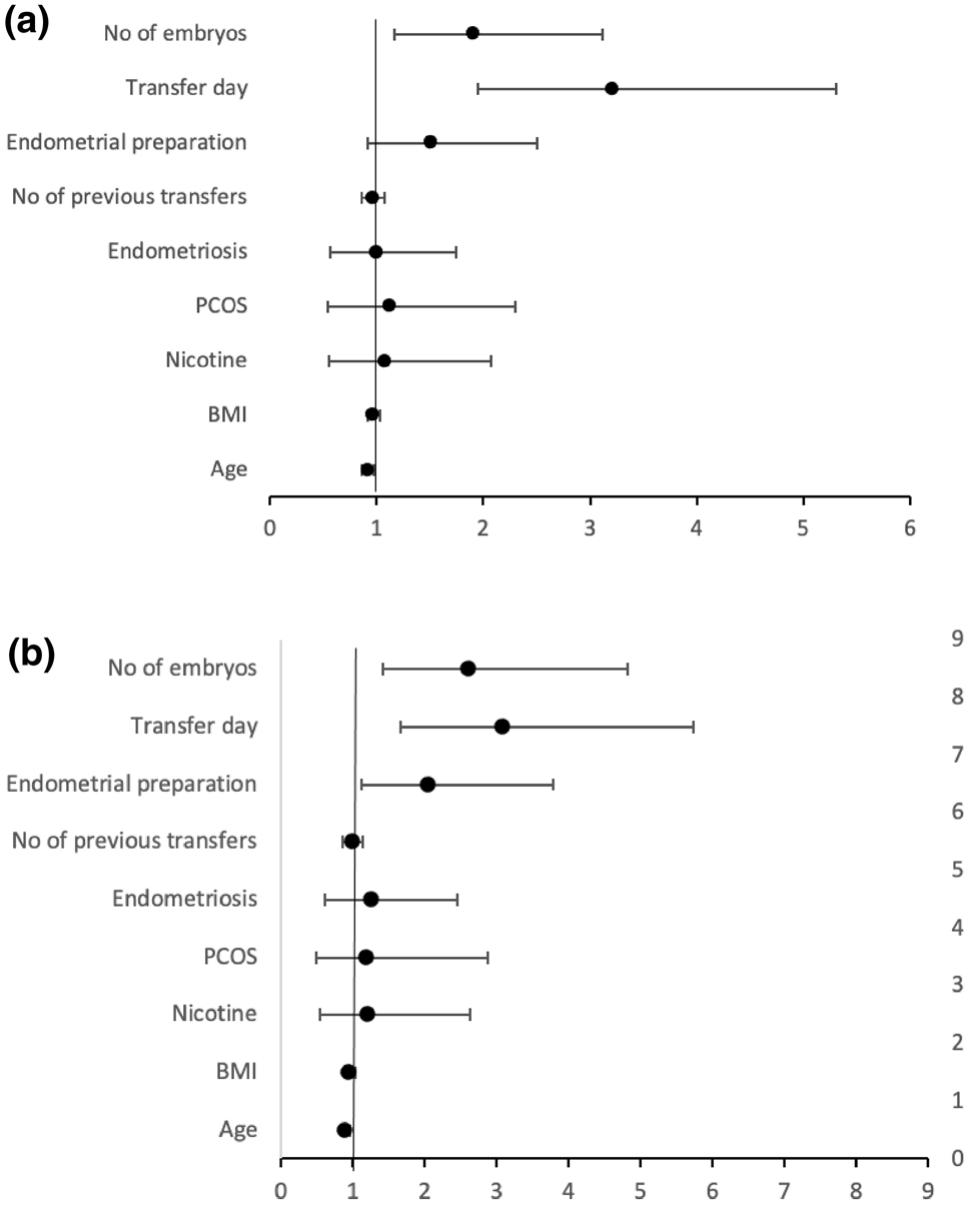
**a** Forest plot of the logistic regression analysis: clinical pregnancy rate as outcome. **b** Forest plot of the logistic regression analysis: birth rate as outcome

In the multivariate logistic regression analysis, the maternal age at the time of the oocyte retrieval, the number of transferred embryos and the culture duration had significant effects both on the clinical pregnancy rate and the birth rate (Tables 3 and 4; Fig. 3a, b). The birth rate was additionally affected by the mode of the endometrial preparation as already seen in the univariate analysis. The time of embryo transfer was the factor with the highest impact on the FET success.

**Table 3:** Multivariate logistic regression evaluating factors influencing the outcome clinical pregnancy rate

Parameter	Regression coefficient	Standard error	*p* value	Odds ratio	95% CI
Constant	2.4739	1.4495	0.0879		
No of transferred embryos (2/1)	0.3224	0.1260	0.0105	1.906	1.163; 3.123
Transfer day (4–5/2–3)	0.5836	0.1280	< 0.001	3.213	1.945; 5.306
Endometrial preparation (mNC/HRT)	0.2097	0.1277	0.1005	1.521	0.922; 2.509
No of previous transfers	− 0.0389	0.0586	0.5068	0.962	0.857; 1.079
History of endometriosis (yes/no)	− 0.00247	0.1431	0.9862	0.995	0.568; 1.744
PCO-Syndrome (yes/no)	0.0547	0.1849	0.7676	1.115	0.540; 2.303
Nicotine abuse (yes/no)	0.0340	0.1693	0.8409	1.070	0.551; 2.078
BMI	− 0.0311	0.0296	0.2931	0.969	0.915; 1.027
Age at oocyte retrieval	− 0.0921	0.0321	0.0041	0.912	0.856; 0.971

**Table 4:** Multivariate logistic regression evaluating factors influencing the outcome birth rate

Parameter	Regression coefficient	Standard error	*p* Wert	Odds ratio	95% CI
Constant	2.7276	1.7650	0.1222		
No of transferred embryos (2/1)	0.4790	0.1564	0.0022	2.607	1.412; 4.813
Transfer day (4–5/2–3)	0.5635	0.1582	0.0004	3.086	1.660; 5.739
Endometrial preparation (mNC/HRT)	0.3604	0.1560	0.0209	2.056	1.116; 3.789
No of previous transfers	− 0.00901	0.0720	0.9004	0.991	0.861; 1.141
History of endometriosis (yes/no)	0.1184	0.1682	0.4815	1.267	0.655; 2.451
PCO-syndrome (yes/no)	0.0888	0.2234	0.6921	1.194	0.496; 2.878
Nicotine abuse (yes/no)	0.0914	0.1994	0.6467	1.201	0.549; 2.623
BMI	− 0.0496	0.0368	0.1773	0.952	0.885; 1.023
Age at oocyte retrieval	− 0.1109	0.0387	0.0041	0.895	0.830; 0.965

## Discussion

Our data clearly show that younger age, double embryo transfer, blastocyst culture, good embryo quality and in older patients additionally endometrial preparation with modified natural cycle (compared to HRT cycle) are positive prognostic factors for ART success. To our knowledge, this is the most comprehensive multivariate regression analysis of prognostic factors of FET cycles published so far.

With an average clinical pregnancy rate of 25.5% the success rate of our FET collective is comparable to the German cPR per transfer of 25.4% in 2016 [20] and slightly lower than the European average pregnancy rate after FET (cPR 29.2% in 2015 [21]) during the same time. In the European comparison the difference is fully explainable by the strict German embryo protection act: In Germany it is prohibited by law to culture more embryos than needed for transfer. The number of cultured embryos must be assessed based on the patient´s age and/or results of previous IVF cycles.

As expected, our patients with a clinical pregnancy after FET were significantly younger at the time of the oocyte retrieval than patients without successful FET. In the multivariate regression analysis, age at the time of the oocyte retrieval is one of the independent factors that affect the pregnancy and birth rate. This finding is consistent with the current literature for fresh transfers as well as for frozen embryo transfers [11, 12] and is explained by the lower oocyte quality and higher aneuploidy rate in older patients.

As in fresh ART cycles, the success rates of FET depend on the number of transferred embryos. In our collective, double embryo transfers in FET cycles resulted in higher clinical pregnancy rates and birth rates compared to FET of a single embryo. In the multivariate regression analysis, the number of transferred embryos was confirmed as an independent influencer of the clinical pregnancy and birth rate, confirming the results of a former study by Veleva [8].

A FET after blastocyst culture is associated with significantly higher clinical pregnancy and birth rates compared to the transfer of cleavage stage embryos, both in the whole collective and in the age-stratified subgroups (Fig. 1), comparable to the results of a Chinese study of He et al. [22]. A limiting factor was the use of two different freezing protocols: Slow freezing was used for PN oocytes and vitrification for MII oocytes and embryos. According the revised ESHRE guidelines for good practice in IVF laboratories [23] vitrification is recommended for MII oocytes, cleavage embryos and blastocysts, but for PN stages good results can also be obtained using slow freezing methods.

In our collective patients with FET in a modified natural cycle had significantly higher birth rates compared to patients with full hormone replacement as endometrial preparation (Table 4). The superiority of transfers in modified spontaneous cycles to HRT cycles in terms of the life birth rate has also been shown in a Finish retrospective non-randomized cohort study of 1972 FET [8] and a multicentric French study [24]. Other studies found no correlation between the mode of endometrial preparation and the FET success [14, 25–27], including the prospective randomized-controlled non-inferiority ANTARCTICA trial from the Netherland [25] and a Cochrane analysis [28]. The main limitation of our analysis with respect to the endometrial preparation is the retrospective non-randomized study design: Patients with chronic anovulation, oligomenorrhea and PCOS were regularly allocated to the artificial cycle FET group. An age-related bias was minimized by stratification into two age-subgroups. But a selection bias due to a higher comorbidity in the older artificial cycle-FET group cannot be ruled out without further investigation of the patient records. At least, the BMI did not differ between the group of mNC and HRT as endometrial preparation (23.7 ± 4.2 in both groups). However these findings should be taken into account in the clinical setting, as a large number of studies have shown an increased risk of preeclampsia, hypertensive disorders and birth complication after HRT-FETs compared to natural cycles, modified natural cycles and low dose FSH-stimulation in recent years [29–32]. The underlying cause seems to be the missing corpus luteum in HRT cycles with missing production of vasoactive substances.

Many studies did not show a correlation of the endometrial diameter with the pregnancy rate in FET [16, 25, 33]. Others found a dependency in their collectives [9, 13, 34, 35]. In our collective, a sonographic endometrial diameter of at least 8 mm in patients younger than 35 years is associated with higher implantation rates compared to a diameter of 7 mm or lower (Table 1). The rate of double embryo transfers and blastocyst culture was comparable between both younger-aged subgroups (44% DET in EMR < 8 mm versus 47% in EMR ≥ 8 mm; 52.8% blastocyst culture in EMR < 8 mm versus 50.0% in EMR ≥ 8 mm).

In order to analyze the influence of an additional poor-quality embryo we compared DET of a good-quality and a poor-quality embryo with a SET of a good-quality embryo after blastocyst culture. While no benefit from performing a DET over a SET in this constellation could be found, -conversely- the addition of a poor-quality embryo to a good-quality embryo did neither have an adverse effect on the clinical pregnancy rate nor on the birth rate over a SET with a good-quality embryo only.

The number of previous transfers, diagnosis of a PCOS, nicotine abuse at the time of the oocyte retrieval and a history of endometriosis did not affect the outcome in our collective; however, the number of affected women within these groups was low. In contrast to the studies of Veleva [8], we could not see any correlation of the FET outcome with the BMI, both in the univariate and the multivariate analysis. This might be explained by the fact that we regularly exclude patients with severe obesity (BMI > 35) from the ART program due to the obesity-associated pregnancy and birth risks. As in fresh ART cycles, an influence of the BMI on the FET success is biologically plausible and may be substance for further investigations.

## Conclusion

In conclusion, we found a significant and independent influence of maternal age, blastocyst culture, number of transferred embryos and the mode of endometrial preparation on the outcome of cryo-embryo-transfers. Together with the recent data about adverse pregnancy outcomes after programmed FET cycles, our analysis contributes to the decision to clearly favor natural FET cycles whenever possible.

## Data Availability

The dataset generated for this study are available on request to the corresponding author.

## Abbreviations

BMI: Body mass index
BR: Birth rate
cPR: Clinical pregnancy rate
DET: Double embryo transfer
FET: Frozen embryo transfer
GQE: Good-quality embryo
hCG: Human chorionic gonadotropin
HRT: Hormone replacement therapy, hormonal substitution
ICSI: Intracytoplasmic sperm injection
IVF: In-vitro fertilization
LFU: Lost for follow-up
LGA: Large for gestational age
MAR: Medically assisted reproduction
mNC: Modified natural cycle
ns: Non-significant
PQE: Poor-quality embryo
sdv: Standard deviation
SET: Single embryo transfer
95% CI: 95% Confidence interval
